# Endangering the integrity of science by misusing unvalidated models and untested assumptions as facts: General considerations and the mineral and phosphorus scarcity fallacy

**DOI:** 10.1007/s11625-021-01006-w

**Published:** 2021-08-26

**Authors:** Roland W. Scholz, Friedrich W. Wellmer

**Affiliations:** 1 Department of Knowledge and Information Management, Danube University Krems, Dr.-Karl-Dorrek-Strasse 39, 3500 Krems, Austria; 2 Department of Environmental Systems Science, ETH Zurich, Universitaetsstrasse 22, 8092 Zurich, Switzerland; 3 Institute for Advanced Sustainability Studies (IASS), Berliner Strasse, 130, 14467 Potsdam, Germany; 4 Neue Sachlichkeit 32, 30655 Hannover, Germany

**Keywords:** Phosphorus scarcity, Integrity of science, Solutionism, Transdisciplinarity, Falsifiability of science, *Faktengewalt*, Truth to power, Consensus to truth, Science activist, Science advocacy, Incompleteness of knowledge

## Abstract

There is increasing demand for science to contribute to solving societal problems (solutionism). Thereby, scientists may become normative activists for solving certain problems (advocacy). When doing this, they may insufficiently differentiate between scientific and political modes of reasoning and validation (de-differentiationism), which is sometimes linked to questionable forms of utilizing the force of facts (German: *Faktengewalt*). Scientific findings are simplified and communicated in such a way that they acquire a status as unfalsifiable and absolutely true (truth to power). This becomes critical if the consistency and validation of the findings are questionable and scientific models underlying science activists’ actions are doubtful, oversimplified, or incorrect. Herein, we exemplarily elaborate how the integrity of science is endangered by normative solutionist and sociopolitically driven transition management and present mineral scarcity claims that ignore that reserves or resources are dynamic geotechnological-socioeconomic entities. We present the main mineral scarcity models and their fallacious assumptions. We then discuss the phosphorus scarcity fallacy, which is of particular interest as phosphorus is non-substitutable and half of all current food production depends on fertilizers (and thus phosphorus). We show that phosphorus scarcity claims are based on integrating basic geoeconomic knowledge and discuss cognitive and epistemological barriers and motivational and sociopolitical drivers promoting the scarcity fallacy, which affects high-level public media. This may induce unsustainable environmental action. Scientists as honest knowledge brokers should communicate the strengths but also the constraints and limits of scientific modeling and of applying it in reality.

**Supplementary Information:**

The online version contains supplementary material available at 10.1007/s11625-021-01006-w.

## Societal relevance, *Faktengewalt,* falsifiability, and the integrity of science

It is the noble task of scientists to advance science by discovering new knowledge in honest ways and through honest processes (Pielke 2007). Good science always questions and critically examines assumptions and attempts to induct a paradigm change (Kuhn 1962). We posit that this may have changed in recent years; one reason is that the general objective of purposefulness, also called social relevance, has strongly shifted from the inner scientific principles of truth and novelty to social usefulness and (societal) solutionism (Strohschneider 2014). With solutionism, we develop restricted conceptions of science that target the private entrepreneur for problem-solving (i.e., capitalizing knowledge; Etzkowitz et al. 1998), or that science must serve to attain the goals of political and/or governmental programs. In Europe, these two solution-oriented views of purposefulness can be distinguished, and both fall under the label of a third mission (Scholz 2017, 2020). Private-good–oriented solutionism (Etzkowitz et al. 1998) is based on contract-based research that often restricts the scientific publication of data and findings; the control of the research is widely handed over to the principal supplying the funds. Moreover, research and some political problem-solving–oriented programs serving the public good are overly constrained by the normative dimension (Zomer and Benneworth 2011). Science becomes functionalized for assisting governmental programs or promoting change processes through specific social innovations; here, scientists become societally accountable (Gibbons et al. 1994; Weingart 1999). In this conception, scientists may even adopt roles as activists or transition-politics advocates (Wittmayer 2016).

A specific peril of this way of conducting science is to fail to distinguish between the roles of scientist and politician (Strohschneider 2014) This may be called the de-differentiation of science and politics. One must be aware of the valuation of uncertainties and the incompleteness of knowledge in the generation and communication of knowledge and scientific models. Additionally, aspects of accountability and responsibility play an important role when communicating or utilizing scientific knowledge. This holds true both in the seemingly negative utilization of scientific knowledge as well as its seemingly positive utilization in programs for sustainable action, e.g., on intergenerational fairness (Brundtland et al. 1987).

The integrity of science is further strongly endangered by a naïve view of science as providing the right or true knowledge. From a realist, normal science perspective, scientific theories are approaching a valid description of complex systems or even of the universe. But, in general, scientific models are generally incomplete. Falsifiability (Popper 1969), the possibility of refutation (Lakatos 2015), and some sceptical undertones (Unger 1978) are basic and essential elements of scientific reasoning and discourse. In somewhat simplified wording, one may say that high-level science may fail. This is also true for the judgments of the Nobel Prize committees; for instance, Nobel laureate Johannes Fibiger’s (1867–1928) theory that worms cause cancer is viewed as refuted (Stolt et al. 2004).

If theories are viewed as indisputable or as truth, the consequence is the evolution of Faktengewalt (engl.; force of facts; Hoffmann 2013; Strohschneider 2014) or Faktenlage (Grunwald 2015). A body of data (facts) and scientific knowledge are no longer questioned because they have become legitimized by some scientific reference. The power of Faktengewalt may be misused conceiving it as “quasi-immunized”. Faktengewalt may also develop a special role in the context of the precautionary principle. Politicians under pressure have to rely on scientific advice. The validity and impact assessment of which may be unclear.

The conviction to always be right has a consequence and may easily become truth speaking to power. The sensibility for the “incompleteness” of science, to be aware not only of “known unknowns” but also “unknown unknowns” becomes lost (Ravetz 1987, 1993). The humble awareness that science creates knowledge but not truth disappears. Seldom are scientific opinions unanimous. This may result in conflict as politicians are used to rely on consensus opinions. Over time, the idea that truth is a matter of majority (e.g., the number of citations in the Web of Science are taken as criterion of validity) makes minority opinions disappearing. Critical, contradictory opinions are necessary to induce paradigm changes (Kuhn 1962). Reviewers outside such a school are not asked for opinions anymore: peer review as “a critical connoisseurship of quality of science” (Ravetz 1987, p. 109) mutates into censorship by peer review.

Under pressure to maximize third-party funding for research, the chances of getting research projects approved can be supported by belonging to the right “school” and by providing results that may be well-aligned to a political strategy and not by being an outsider. Among the general public then, the opinion takes hold that “science” is not “science” anymore but an undisputed body of facts. The history of science may serve as an example. The opposite view of giving up science as a reference system has been promoted by post-normal science. Here, science becomes one voice among others. The objective of science to approach reality by approaching rules that are at work in natural and/or social systems by measurement (Durkheim 1895/1982) has been relinquished.

This paper deliberates how the above-mentioned features of science may affect the integrity of science. We will do this, particularly, in relation to fallacious statements on the scarcity of minerals (based on the fixed stock assumption underlying mineral scarcity statements) using the example of phosphorus, an essential, non-substitutable element for life and food production. About half of all current food production is based on primary mineral fertilizers (including phosphorus) and, thus, on rock phosphate as the ore (Scholz et al. 2014).

## Concerns, the normative and the real

Applied research is of interest to the public and to politics, but scientific knowledge and technologies are often Janus-faced, with ambivalence and ambiguity linked to them. How to use scientific knowledge depends on the context and the intentions of the user and is generally linked to the incompleteness of scientific modeling. This is particularly true if new views are communicated that impact key political and/or economic actors and the knowledge is picked up and conveyed by mass media.

Science researchers address and intermingle with non-scientific institutions, politics, lobbying groups related to the economy, etc. as scientific actors. This aligns with the rule that, if the stakes have strong links to the impacts of scientific statements, the scientific debate becomes politicized (Jasanoff and Wynne 1998). Environmental sciences and climate research may be taken as examples of transformative science where scientists describe, deliberate, and push political action through urgent calls for change (Grunwald 2015; Schneidewind 2015; Strohschneider 2014; WBGU 2011). “Transformation science explicitly addresses values and envisions desirable futures” (Tschakert et al. 2016, p. 21). This may induce two different pathways. On one hand, the decisions remain under the responsibility of practice and politics. This pathway may induce mutual learning between science and practice where both sides remain in their roles and launch transdisciplinary processes (Scholz 2017; Scholz and Steiner 2015; Scholz et al. 2021; Wittmayer and Schäpke 2014). On the other hand, it may cause scientists to become solutionists for fixing social problems in the same way as politicians.

### Shallow action research: Scientists’ values at work

Curiosity, arising “from the perception of a gap in knowledge or understanding perception” (Loewenstein 1994, p. 78), has been viewed as a key driver of scientific activity. Interest in a particular topic is another. But social normative concerns and value-based goals are also drivers. In line with John Dewey’s pragmatism, Kurt Lewin, as a director of MIT’s Research Center for Group Dynamics, the father of social psychology founded experimental action research to combine science and social practice (Adelman 1993). Lewin’s personal interest in addressing the problems of minority groups led to a problem-oriented collaboration of science and practice that applied scientific methods and involving practitioners in the process (i.e., participatory research). The basic idea of equal rights for all people was the overarching societal concern of Lewin’s research. Which course and action was considered the most effective was not defined in advance, but, instead, jointly developed with practitioners, and the reasons and options were explored and experimentally tested.

Lewin’s approach differs from certain types of action research in which scientists’ (intuitive) opinions about how the real world should work (in contrast to reality or other interest groups’ goals) and not the investigation and testing of the processes and mechanisms causing a certain state are essential. Action research that is strongly dominated by a scientist’s personal objectives on what reality should be is denoted as shallow action research (Dedeurwaerdere 2018; Scholz 2011). Societal and sometimes personal norms rather than hypotheses (on them) are guiding research. Science should serve to create a better world according to specific goals and norms of groups of scientists. This is often done when taking a “post-normal science and action orientation” perspective which “is normative” and aims “to influence transformative changes while studying them” (Loorbach 2014, p. 68).

This approach has three main features. First, specific goals related to how the world should change are explicitly or implicitly defined by scientists. Thus, scientists become politicians by defining the norms that, in democratic societies, are negotiated among stakeholder groups. This includes the value-based selection of practitioners whose participation is not based on a societal stakeholder analysis which includes, e.g., representatives of key actor groups which are concerned by, causing, or regulating a critical problem. Advocacy science activists often include only those stakeholder groups that meet the scientists’ social values. For instance, one may include only those who explicitly declare themselves to be committed to sustainability or decide to collaborate with those stakeholders who consider themselves as working for and with the poor (Rosendahl et al. 2015). This leads to, second, a principal value-based exclusion of certain stakeholders. This violates the rules that science, in democratic societies, has to serve all societally stakeholder groups whose action are compatible with the human rights and the national constitution and to include that stakeholder groups which are necessary in order to understand transition processes.“

Naturally, there are delicate ambiguities involved in the question of which stakeholders should be included. Think about members of an extreme radical party being under the supervision of national intelligence, yet part of a democratic parliament. This shows that there are no general rules for all situations, governments, countries, etc. from an integrity of science perspective. Finally, third, we may find the proposal that the best and highest form of transdisciplinarity would result if there were to be “joint decision making. The idea of concerned science is to contribute to the generation of normative and action-guiding knowledge, in particular” (Wiek 2007, p. 55). This may be viewed as a variant of postmodernism’s zeitgeist and Faustian dream referring to science as a source of societal concern that legitimizes participating in any relevant societal decision.

In addition, we wish to note that science certainly plays a special role as a kind of clearinghouse of knowledge (Scholz 2011) in the sense that the validity of statements is judged with transparent criteria and methods, thereby revealing value-oriented assumptions. Science and education are seen as basic pillars and public goods of society serving all stakeholder groups. Shallow action research is in contrast with transdisciplinary processes in which scientists do not work as normative science activists but, rather participate in mutual learning processes between science and practice that relate (a) targeted interdisciplinary knowledge (which allows for a better understanding of complex problems) with (b) normative and interest driven multistakeholder processes (Scholz 2020; Scholz et al. 2000; Scholz and Steiner 2015).

### Concerns about limits of resources

*The development of the scarcity fear and the fixed stock/pie assumption*: Subsequently we discuss how the availability of raw materials has become an object of science activists’ normative venture when utilizing Faktengewalt of unquestioned and wrong scientific statements. The availability of raw minerals is an important and complex issue. The cultural and technological development of humankind has been possible only by using mineral resources. Therefore, our technological development has been labeled according to raw materials: the Stone Age, Bronze Age, Iron Age, and now, what many call the Silicon Age. Since man was able to care for tomorrow he probably thought sorrowfully about future resources supply, a concern which can be traced through ages. Stone Age man will have worried about where he might find the next suitable piece of flint. His search was not only a question of finding it but also a question of economic competition and survival because others sought the same good-quality raw material. The British economist Jevons predicted a shortage of coal in 1865 (Jevons 1865). Today Britain still has large untouched coal resources, the use of coal being replaced by cheaper fossil fuels oil and natural gas. In 1938, Australia banned all iron ore exports because of fear of exhaustion, a ban that lasted till 1960. Today Australia is the largest iron ore producer and exporter of the world (Lee 2013). Unfortunately, based on everyday experience that the pie on the table has a fixed size (Bazerman et al. 2001), a “mythical fixed-pie mentality” (or the fixed stock paradigm; see, e.g. Tilton 2003) became a prevailing misconception and bias. This conservative precautionary bias is based on supply fear inherent in some cultures (Radin 1937): When and where will I get the necessary raw materials? Will I get them at all? After World War II, environmental awareness grew in industrialized nations and created another problem, the sink problem.

An excellent example of this “source and sink approach” was the highly influential book by Meadows et al. (1972a, b) titled *The Limits to Growth: A Report for the Club of Rome’s Project on the Predicament of Mankind*. The Club of Rome was founded in 1968 by a number of high-level politicians, business leaders, and scientists who were concerned by the exponential growth in world population and its corresponding resource consumption. Meadows et al. (1972a, b) were early masters of dynamic systems modeling related to Forrester’s world dynamics model (Forrester 1969, 1971). They considered the limited resources as a foundational case as well as the pollution resulting from an exponential use of resources. As a result of their world model, it was postulated that scarcity of natural mineral resources and environmental degradation would lead to serious crises before 2100 and to a collapse and return to simple living conditions.

**Box 1:** Critical assumptions of limit of growth

In The Limits to Growth book, Meadows and colleagues (1972a, b) stated: Even given high prices, silver, tin, zinc, and uranium may become scarce before the year 2000. We expect that, given the current consumption rate, the deposits of other minerals will be depleted (Meadows 1974, p. 55). Meadows et al. also made reference to geology when stating that whether there will be unknown deposits is disputed. Thus, it would be unwise to rely on newly explored sources. Yet, if we take the example of zinc, in 1970, the ratio of reserves to production was 23. Today – a half-century later – it is just slightly lower at 19. This is practically the same, despite of a 2.3 times increase of production from 5.6 million tonnes in 1970 to 13 million tonnes in 2019. Over the 50 years between 1970 and 2019, a total 429 million tonnes of zinc were produced. This is 3.5 times the reserves known in 1970 to Meadows et al. (1972a, b). Meadows et al. acknowledge exploration as a dynamic variable, with the outer limit as the enlargement of the reserves by a factor of five. This proves overly pessimistic. The resources at present is 1.9 billion tonnes, i.e., 7.6 times the present reserves and 15.4 times the reserves of 1970 according to Meadows et al. Clearly, this example demonstrates that reserves are a dynamic quantity.	

**Fig. 1:**
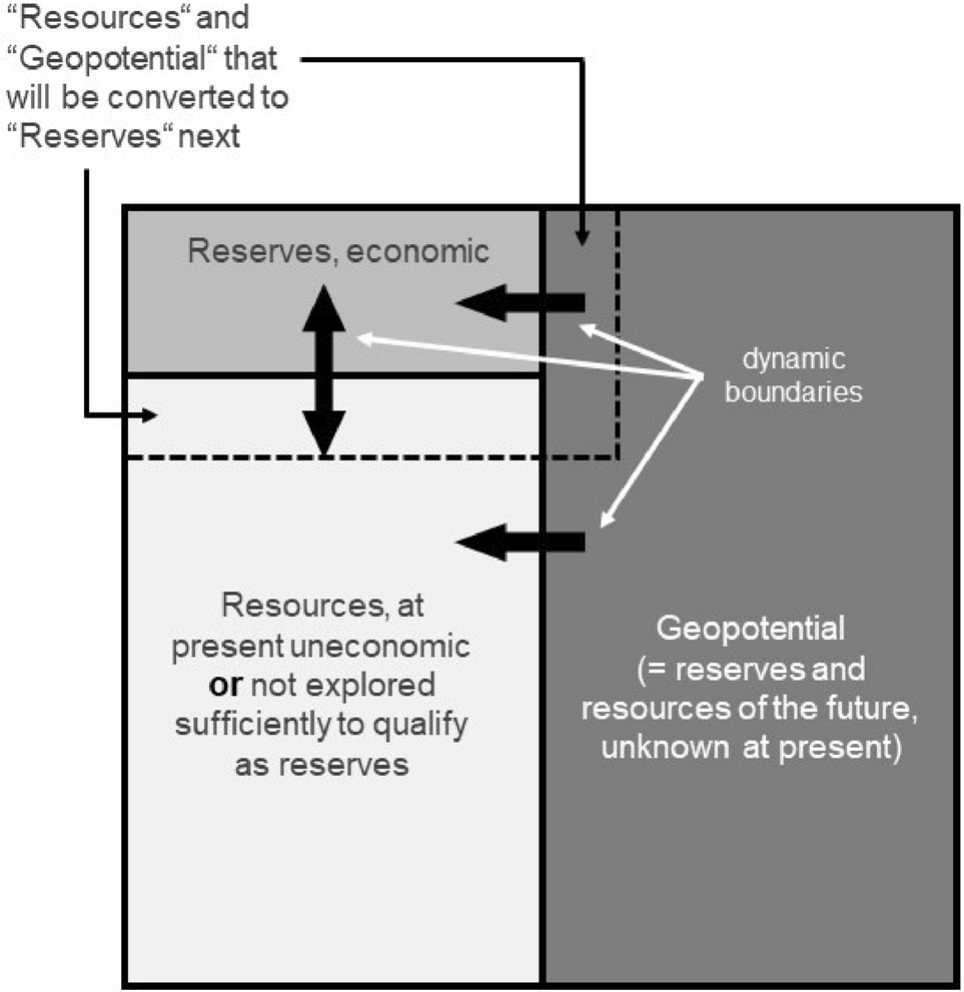
The Total Resource Box (Scholz and Wellmer 2013); *x*-axis: general trend of increasing geologic knowledge, going from right to left; *y*-axis: general trend of increasing economic viability, going from bottom to top

The Meadows et al. (1972a, b) Club of Rome report influenced the thinking of many people. It resulted in a scarcity fear and stimulated a huge boom in the exploration for minerals in the 1970s. Thus, we will scrutinize their approach concerning the availability of natural resources. Meadows et al. conceived everything as being dynamically, except for the reserves and resources. The reserves represent the amount of known and assessed mineral commodities that can be mined economically with the present prices and technologies under environmentally and socioeconomically acceptable conditions. The resources are known (at various levels of certainty), but their economic viability has not been established or their grade is too low and requires better technology or higher prices to become economically feasible. Thus, the resources may include, at a certain point in time, “economic, marginal economic, and subeconomic components” (USGS and USBM 1980, p. 1). And reserves and resources are only a small subset of “all there i*s*” as will be explained below with Fig. 1 (Meinert et al. 2016). It is surprising that mineral reserves and resources were viewed *not* as dynamic quantities. It is easy to infer that a higher price allows mining lower grades of an ore body (Wellmer and Becker-Platen 2002). And there is a general geological experience that the total amounts of additional reserves and resources are increasing non-linearly and disproportionately high with lower ore grades. For frequently in nature observed logarithmic grade distributions the following applies (Lasky’s rule): A linear decrease in grade is accompanied by an above-linear (non-linear) increase in cumulative tonnage of the ore (DeYoung 1981).

Although Meadows et al. (1972a, b) take into account certain dynamic elements such as successful exploration up to a certain limit or by incorporating subeconomic resources, in general, they ignore the market mechanism of prices regulating the supply and demand of raw materials via the economic feedback control cycle of mineral supply, thereby neglecting to consider a basic rule of the geoecology of available resources (Scholz and Wellmer 2013; Wellmer and Dalheimer 2012). We are facing a seeming paradox that prices, technology development, exploration, and other factors increase reserves and resources. Thus, reserves grow with consumption. Box 1 demonstrates this for zinc. With an increase in consumption, production has to increase, therefore revenues and in most cases also profits. This enables to intensify exploration activities and to increase the chances of finding additional reserves. Mining companies want to keep a balance between reserves and production for planning purposes. Besides extension of known and discoveries of totally new deposits orebodies by exploration there are two additional effects which help to keep the balance between reserves and production: (1) In case of commodity prices increase, the cut-off grade of the mining operations can be lowered, thereby lowering the average grade with the consequence of increase of tonnage as just described (DeYoung 1981; Lasky 1950b; The Bureau of Mines 1974). There is a rule of thumb in practice that halving the cut-off grade will increase the ore tonnes by the factor of 4 and the contained metal by the factor of 2 (Schodde 2019). (2) Average grade can also be lowered due to technological learning processes and then the increase in tonnage and metal content again is the consequence. As an example, the world-wide copper grade in 1970 was about 1.65% Cu; today (2018) it is 0.56% Cu (Schodde 2019).

The second motive for assuming raw materials must be scarce may be related to the experiences of past generations that faced real scarcity during WW I, the Great Depression years of the 1930s, WW II, or – much later – the 1973 and’79 oil crises and short-term price crises when everyone experienced the impacts of scarcity. Scarcity fears were not reasoned and could be observed in the recent coronavirus pandemic when people began to hoard items that were necessities of daily life (Garbe et al. 2020). There are strong people’s desires to live in a perfectly secure world (Daston 2020), underlined by the growing numbers of insurance policies. Together with the growing awareness of sustainable development and the commitment for intergenerational equity, it becomes understandable why the scarcity fallacy of mineral resources and the underlying fixed-stock assumption became a no longer critically examined Faktengewalt (Schmidt 2019) and why it finds its way into publications, submissions for research projects, newspaper articles, etc.

## Models of mineral scarcity and dynamics of resources/reserves

We now explain why raw materials are not scarce and why the fixed-stock assumption is false. The answer requires an understanding of some geological concepts.

### The dynamics between reserves, resources, and geopotential

When looking to the future, a third category besides reserves and resources (defined above) must be introduced. By means of modern exploration technologies, future, so far unknown reserves and resources, the geopotential, may be discovered from the Earth’s crust (see Fig. 1).

Reserves are typically assessed by mining companies. They are discovered in the “reservoir” of the geopotential or are transferred from the resource field of Fig. 1 by an increase in market prices. This increase enables higher exploitation costs and/or improvements in technology can make it possible to mine lower-grade ores. Reserves serve as a company’s planning data. Their interests are in those reserves that can be mined within their planning horizon, which normally ranges from 30 to 100 years. Expensive data for longer time ranges for reserves are not of interest. However, mining companies are interested in maintaining a balance between production and reserves. Therefore, reserves grow in parallel with increasing consumption. The example of zinc is presented in Box 1. Another example is crude oil. In 1950, crude oil reserves were 11.3 billion tons and production 543 million tons provided a reserve-to-production ratio of 20. In 2018, 68 years later, the reserves were not exhausted but had grown by factor 22 to 244 billion tons at a production of 4.5 billion tons and an increased ratio of 55. These time series of the ratios between reserves and production clearly show that the fixed-stock concept is erroneous.

The dynamic boundary between reserves and resources in Fig. 1 is primarily determined by price and technology. With higher prices and better technologies, resources can become reserves, and the opposite happens when prices deteriorate. Since future commodity prices are unknown, the future boundaries cannot be predicted accurately. The concept of reserves is not a physical but rather a geoeconomic one (Kooroshy et al. 2009; Tilton et al. 2018). Thus, reserve numbers change continuously due to economic conditions, technological changes, further exploration, etc. The published numbers are simply a “snapshot” of a highly dynamic system (Wellmer 2008) and certainly no indication of “all there is.” This is acknowledged by the US Geological Survey scientists (Meinert et al. 2016) or by the German science academies (Leopoldina 2018).

### Approaches for modeling mineral scarcity

We present the main approaches for modeling prospective global short- and mid-term scarcity. All of them rely on erroneous assumptions for modeling ultimately recoverable resources (URR). We examine the reserve-to-production ratio, the peak production theory, and the assumption of being able to estimate the URR and the abiotic depletion potential.

#### The reserve-to-production ratio (R/P ratio)

The attempt to forecast the availability of raw materials using the ratio of reserves to production (the R/P ratio) had already begun by the middle of the 1800s. An example is Jevons’s (1865) study on coal availability in Great Britain. In the mid-nineteenth century, the leading nations founded geological surveys in order to map their territories for discovering natural resources as a basis for industrial development. These geological surveys also started to keep records on production and reserves to the extent that these were publicly available. Yet, the geological surveys, with a good understanding of the dynamic nature of reserves, did not publish a ratio of reserves and production (presumably as it has been too often misunderstood). Today, the most frequently quoted publications concerning reserves and production are the Mineral Commodity Summaries (MCS) of the US Geological Survey (USGS), based on the research of many commodity and country specialists. Unfortunately, the R/P ratio is continually misinterpreted by non-economic geologists and non-resource scientists to be the “lifetime of reserves”. This wrong interpretation promotes the emergence of the fallacious mineral scarcity fear. The ratio of reserves to production is a dynamic figure and does not mean a lifetime. It is, instead, a snapshot of a dynamic system (Leopoldina 2018).

The R/P ratio is unsuitable as an indicator of lifetime. It rather can be used as an indicator of the need for exploration (Wellmer and Becker-Platen 2002). It may serve as an early warning indicator if the ratio consistently falls instead of remaining constant or increasing in a certain range (Scholz and Wellmer 2013). Commodities with lenticular ore deposits like the deposits of precious and base metals such as copper or zinc have R/P ratios between 10 and 40. Continuous exploration efforts are necessary to maintain mining companies’ balances of the reserve-to-production ratio to guarantee supply security. Commodities occurring in layers continuing over long distances and stratiform deposits with R/P ratios larger than 100 do not need constant efforts of explorations, because these time periods are mostly beyond the planning horizons of mining companies.

#### The peak model of production and the assumption of the ability to estimate ultimate recoverable resources (URR)

The peak model uses the observation that the production lifetime curve of a confined area, like a single mine or a mining country, frequently follows a bell-shaped curve. This was first applied to forecasting by Hewett (1929), then by Lasky (1950a), and later by Hubbert (1956). M. King Hubbert used it apparently successfully in 1956 to predict the peak of US oil production (lower 48 states) in 1971 with an error of only one year. Because of its seeming exactness, the bell-shaped curve known as the Hubbert curve became famous. This peak concept has since been applied by many authors to forecast limits of availability, for example, for phosphorus by Cordell et al. (2009), to be discussed later; by Calvo et al. (2017) for a range of commodities; and by Ali et al. (2017), who took copper as an example for commodities generally.

**Fig. 2:**
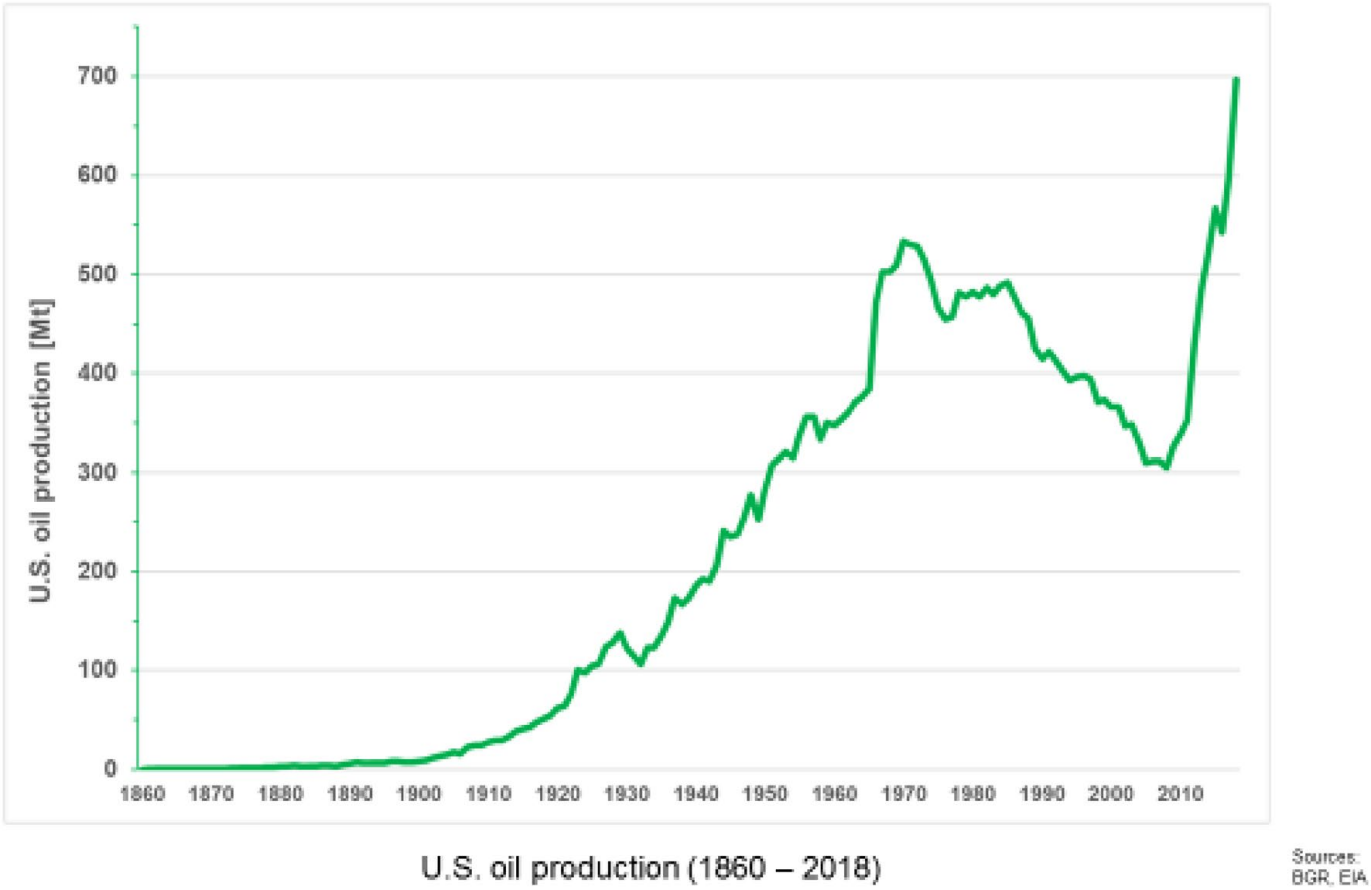
US oil production 1860–2018 (source BGR databank)

The ultimate recoverable resources (URR) refers to the amount that has been mined in the past plus the amount to be mined in the future. Therefore, URR is dependent mainly on future price development and, thereby, the future demand-and-supply situation and technological development. No one can forecast it. The philosopher Karl Popper (1969) claimed that, for radically new innovations to occur at all, the future must be unknowable. Otherwise, an innovation would, in principle, already be known and would occur in the present and not the future. The US hydrocarbon industry is a worthwhile example of how radically a formerly not envisioned technological development, in a Popperian sense, can change the URR. The recently developed technologies of fracking (hydraulic fracturing) and horizontal drilling have made it possible to exploit primary shale deposits, i.e., the hydrocarbons are still at their original place and not mobilized and stored in overlying porous reservoir rock (Tilton 2018; Wellmer and Scholz 2018). With this technology for mining unconventional oil deposits, in 2015, the US overtook the famous peak of 1970 forecasted by Hubbert in 1956 and became the largest crude oil producer in the world in September 2018 (Fig. 2).

Innovation researchers distinguish between incremental innovations and disruptive (or “jump”) innovations (Harhoff et al. 2018). Disruptive innovations are totally new products, processes, technologies, or business models and they replace older ones. Good examples include the invention of transistors replacing tubes and digital photography replacing silver film-based photography.

Such unforeseen technological developments also make it impossible to extrapolate production curves too far out, and certainly make it impossible to estimate URR from historic production. There exists an assumption that, from the increasing part of the bell-shaped curve, the peak and decline can be estimated, a method called Hubbert linearization. This concept has been tested. Rustad (2012) examined 37 production histories of commodities including the development of US and worldwide oil production mathematically (for oil update see Wellmer and Scholz 2017). He demonstrated that the Hubbert linearization process for forecasting production development does not produce realistic results and phrased the title of his paper simply “Peak nothing.”

#### The abiotic depletion potential (ADP)

The life-cycle assessment (LCA) science community uses the abiotic depletion potential (ADP) as a characteristic value to assess environmental cost of material consumption. For the material input into the economy, they want to have a reference value from a source. This has unfortunately developed into an argument for fixed stock and scarcity concerns, too. The question of what kind of “reservoir” is taken arises. As described above, reserve is unsuitable because it is a snapshot of a dynamic variable. The same is true for “reserve base” – a broader term used by the USGS and the now-defunct US Bureau of Mines for an “identified resource that meets specific minimum physical and chemical criteria... [and]... those parts of the resources that have a reasonable potential of becoming available economically within planning horizons beyond those that assume proven technology and current economics” (USGS 2020). In principle, the reserve base shows the same type of dynamics as reserves and resources. The ideal “reservoir” number would be the ultimately extractable reserve (van Oers and Guinée 2016) or the extractable global resource (Drielsma et al. 2016). This is the equivalent of the URR above and, as outlined, impossible to predict due to the imponderability of technological change. Consequently, a reliable database was chosen as a proxy. This is the crustal content as the upper-most boundary possibility and, in the LCA community, is called the “ultimate reserve” (van Oers, De Koning, Guinée, & Huppes, 2002; van Oers and Guinée 2016). The ADP can be accepted as purely scientific (geologic) reference value. However, it becomes problematic when used in criticality assessments, because then crustal values are connected with reserves and implying a defined crustal values to reserves ratio which is a fallacy (Berger et al. 2020; Rankin 2011). This was attempted in defining the list of critical raw materials for the EU in 2010 (EC 2010, p.29/30, Box: Case study: environmental impacts of raw materials). Thereby, a scarcity fear and a fixed-stock concern that have no justification, as shown by Tilton et al. (2018), is fueled.

That the crustal content is also an unsuitable database to estimate the extractable global resource (EGR) (Henckens et al. 2014, 2016) has been shown by West (2020). How unrealistic it is to use the crust as the ultimate reservoir has been calculated by Tilton et al. (2018): Given current states of extraction for copper and using the Earth’s crust as the ultimate resource results in 84 million years, way beyond the most probable existence of man. Tilton, of course, cites this example only to prove that it is not the physical existence of a raw material that is important but the cost for extracting and converting it into a useable product.

## The phosphorus scarcity case

### The fixed-pie approach

Phosphorus is a bioessential element with no substitute (Scholz and Wellmer 2018; Wolfensberger et al. 2008). Other bioessential elements (e.g., nitrogen and potassium) are practically unlimited resources as the they are economical accessible in air and seawater (Lahman and Lassiter 1972). This is not the case for phosphorus. These are the main reasons that the European Commission has ranked rock phosphate among its critical raw materials. i.e., as a mineral with high supply risk and large economic importance (EC 2017) since 2014. In a recent evaluation of criticality studies from 2008 onward, nine studies focusing on phosphorus were identified (Schrijvers et al. 2020). Of these nine studies, six ranked phosphorus as being of high criticality, two as medium, and only one as being of low criticality. The basis of the call for governance and government interference is an example of the unquestioned Faktengewalt. Criticality relates to economically critical short-term supply risk and asks for political action. Claiming an overall physical mid- or long-term scarcity opens a different dimension. A physical mid- or long-term scarcity of mineral phosphorus would challenge assumption of sustainable development (Brundtland et al. 1987) would ask for political action to ensure intergenerational justice.

The present phosphorus R/P ratio stands at 288 (Jasinski 2020). It grew from 90 in 1988 and is higher than for nearly every other commodity. Therefore, the USGS noted: “there are no imminent shortages of phosphate rock” (Jasinski 2020). There is a high level of reliability in these statements that is, affected by uncertainties of production data in two countries, i.e. China and Peru (Geissler et al. 2018). The world’s resources are estimated at more than 300 billion tons (Jasinski 2020). In addition, that phosphate is a low-cost commodity (each person worldwide consumes phosphorus at a cost of under USD $5.00 annually) must be taken into consideration. This leaves room for adjustments to higher prices and, therefore, to mine economically lower grades by lowering the cut-off grade, and to benefit from nonlinearly and disproportionately high reserves growth as described above. Of course, these numbers can always be critically examined in scientific disputes as done, for example, in 2013 and 2016 by Edixhoven et al. (2013) and Scholz and Wellmer (2016). But everyone concerned with practical problems of raw material supply will share the opinion of one of the world’s leading consultants concerning the world’s phosphate inventory, who in 2016 stated that, “… reserves and resources are so large that the term ‘reserves’ has little relevance to the debate over long-term phosphate rock (PR) accessibility” (Mew 2016), certainly in a range of 1000 years. Van Vuuren et al. (2010) reached the same order of 1000 years with their static “additional resources” model that supplements reserves when focusing variation of the demand function.

It has been shown that no sound basis exists for a short- and mid-term raw materials scarcity fear and, especially, none for a phosphorus scarcity fear (e.g., Heckenmüller et al. 2014). But as noted, doomsday forecasts have flourished throughout history. Phosphorus lends itself to such forecasts because without phosphorus for growing food, humankind would perish. People think that economically mineable phosphate will run short because phosphate rock is finite. Thus, imagining a fixed pie, many people are afraid of a rapid end of sufficient phosphorus supply. This basic scarcity fear came to the fore again in September 2019, when the British newspaper *The Guardian* published an article with the headline “Phosphate fertiliser ‘crisis’ threatens world food supply” (Carrington (2019), based on an article by Blackwell et al. (2019)). In a search for sensationalism or attention, “news” of a possible supply disaster appears in waves every 10 to 20 years. This recurring concept of phosphate scarcity (Ulrich and Frossard 2014) can be traced to a 2009 article in *Nature* about phosphate as a “disappearing nutrient” (Gilbert 2009) and further back to President Franklin D. Roosevelt’s 1938 address to Congress on “Phosphates for Soil Fertility” (Roosevelt 1938). In between, we can point to a “cause célèbre” (Brooks and Andrews 1974) of the 1970s – the 1971 Robert Inger report, *Man in the Living Environment* (The Institute of Environment 1972), or to above cited publication by Cordell et al. (2009). These reveal that the above-described thinking of those who refute previous “disaster alarms” – often experts and phosphate insiders – are not taken into consideration (Emigh 1972; Mew 2011; Scholz and Wellmer 2013; van Kauwenbergh 2010).

The highly cited paper in *Global Environmental Change* by Cordell et al. (2009) initiated the most recent scarcity fear. The authors simply took the USGS 1999 reserve data. To them, the URR is just the sum of the historical production (consumption) of mineral phosphorus plus the reserves. To this fixed stock, Cordell et al. (2009) applied the Hubbert curve model, which uses a symmetric logistic model (they apply a Gaussian curve). Thus it “results in a production at peak of 29 MT P/a and a peak year of 2033” (2009, p. 298), summarizing that “phosphate rock … may be depleted in 50–100 years” (2009, p. 292). As a reaction to this wrong statement, the authors of the present paper submitted and published a paper in the same journal (a) explaining why the fixed-pie statement is erroneous and (b) providing an estimate of phosphate rock reserves and resources (given that prices will increase and mining technology improve) of the US Western Phosphorus Field. The assessment resulted in an “estimate..., of the magnitude of 1000 years for static lifetime ‘at most manageable costs’” (Scholz and Wellmer 2013). From a sociology of science perspective, it is interesting why the reception of phosphorus scarcity fallacy message was not really affected by this paper. From a geologic, geoeconomic, and mathematical modeling perspective, the conclusions drawn by Cordell et al. (2009) are fundamentally flawed and incorrect. The modeling with a fixed resource stock would not have passed peer review if experts in mineral resources had been included. The Cordell et al. conclusion falls behind even Meadows et al.’s assumption (see Box 1) of the estimation of the URR when including a growth factor of five.

Thus, it is amazing that the wider scientific environmental community has failed to take note of the refutation of experts. The Scholz and Wellmer (2013) paper was submitted, most thoroughly reviewed, and appeared in the same high ranking journal as Cordell et al.’s (2009) scarcity model. However, the phosphorus short-term scarcity argument has continued to be promoted. This can be demonstrated by a web of science survey (see Table 1). The science scarcity discussion started after the Déry and Anderson (2007) and Cordell et al. (2009) papers. An increasing number of science paper referred to alarmist physical phosphorus scarcity claims (see, Line 3). 10 out of the 14 papers on phosphorus predict rapid depletion.

**Table 1:** Frequency of Web of Science papers on global phosphorus (1) discussing potential scarcity or (2) explicitly mentioning “scarcity/scarce” in connection with resources and/or reserves, (3) explicitly providing an alarmist scarcity statement and (4) papers on scarcity

	Topic	2000/1999	2005/2004	2010	2015	2020
1	Papers on global phosphorus reserves/resources	2 (13)	5 (31)	16 (37)	24 (30)	14 (50)
2	Potential scarcity explicitly discussed (term “scarcity”appears)	0 (0%)	0 (20%)	4 (25%)	9 (41%)	8 (57%)
3	Explicitly referring to “rapid depletion” or “50–150 year” like depletion alarmist statement (share of category 2)	0 (0%)	2 (40%)	9 (56%)	11 (46%)	10 (71%)
4	Number of papers which fulfill criteria of Line 2 *and/or* Line 3	0 (0%)	2 (40%)	9 (56%)	16 (67%)	14 (100%)

How the incorrect use of scientific data may induce fear by Faktengewalt has been demonstrated by the September 2019 *Guardian* article. It argues: as global phosphate consumption increased by 14%, the ratio of reserves to production fell from 300 to 259. This decrease was interpreted as an alarming sign. The ratio for phosphate with 258 is very high. From a mineral resources science view, it is in the order of 15 to 20 times distant from any levels that can be considered critical (Wellmer et al. 2018) with respect to developing mining capacities. For metals, the normal range of the reserve/production ratio discussed above varies, for example between 18 for zinc to 70 for iron ore or 100 for bauxite the raw material for aluminium. We may note that the geopotential offers two other phosphate sources: deep sea phosphate mining and phosphorous from sea water, the first restricted today by environmental, the second by technological problems – unknown knowns which might be solved in the longer-term by human creativity (McKelvey 1972).

It is most interesting to see that phosphorus experts from geological surveys or mining companies, etc. consider the phosphorus scarcity claim as basically wrong (see Table 2, No. 1). These people work at the direct interface with science and usually know the scarcity discussion and the key papers.

**Table 2:** Four questions (see Supplementary Information 1) on practice experts’ agreement related to the “phosphorus scarcity soon” claim (*: This question included a note that “basically wrong” means that an adjustment of the specific period, e.g., by updating reserves, is comprised; ** three experts, not from the field of resources management, explained that the statement is basically wrong, but there will be some point of depletion in the forseeable future)

No	Statements	Ratio of agreement to disagreement of 16 high-profile phosphorus practice experts (statements 2–4 are missing some values)
1	“Phosphorus depletion in 50–100 years” is “basically wrong.”*	15:1 [12:1]**
2	There has been an increasing bias in the last 20 years toward citing papers claiming scarcity	12:2
3	The “phosphorus scarcity soon” argument has influenced the political agenda	12:4
4	Do you know peers, whose belief in integrity has been endangered by the phosphorus scarcity statement 1?	7:5

### Phosphorus practice experts thinking on phosphorus scarcity

Do high-level phosphorus experts follow the phosphorus-scarcity fallacy? To investigate this question, we conducted a survey including the following inquiries: (1) whether current global reserves might be depleted in 50–100 years (per Cordell et al. 2009); (2) whether there has been an increasing bias in the last 20 years toward citing papers claiming scarcity; (3) if and how the “phosphorus scarcity soon” argument has influenced the political agenda; and (4) whether they know colleagues whose scientific integrity has been endangered by the phosphorus-scarcity statement (1)? (For the exact wording of the questions, see Supplementary Information 1.)

We took the phosphorus practice representatives listed in the 2013 organigram of the four-year transdisciplinary Gobal TraPs project (Scholz et al. 2015, p. 78) as a reference sample of phosphorus practice experts. The sample included experts from all links of the phosphorus supply chain: exploration (e.g., geological surveys members), mining (mine representatives), processing (e.g., fertilize-production experts), use (e.g., fertilizer experts), dissipation and recycling (e.g., recycling companies and Greenpeace), and trade and finance (e.g., one CEO of the four largest phosphorus-trading companies). As two of the six specialists from exploration and mining were among those whose current affiliation/address could not be identified, we substituted senior members of the French and US geological survey; 16 of the 22 experts responded.

Table 2 shows that all but one expert (among six geological experts) considered the fixed-pie, peak-modeling-based depletion/scarcity statement as “basically wrong” (see [1], Table 2). The deviating answer was provided by an expert in biological agriculture. Three of the respondents remarked that the time frame is wrong, indicating that they see some point of depletion (perhaps in relation to a fixed-pie model). The vast majority of experts agreed in regard to the citation bias related to scarcity (see question 2 in paragraph 1 above) and that the scarcity argument has been used to affect the political agenda (see question 3). The majority of experts (58%, see question 4) know people whose integrity in relation to science has been endangered by the statement (1).

### Alternatives to the fixed stock/pie concept

The Earth has limits. Therefore, the URR of non-renewable resources must have limits. Thus, for the proponents of strong sustainability (state of non-diminishing life opportunities according to Daly and Cobb 1989), the minimum or non-use of non-renewable resources is the only solution. There are alternatives, however, in the form of two approaches that strongly rely on humankind’s creativity (McKelvey 1972) and the influence of price on reserves. The first relates to *non-*bioessential materials. For such materials, humankind does not need raw materials, as such, but solutions to functions. Therefore, the alternatives of substitution exists, either directly or by technology (Wellmer et al. 2018, p. 90). The second solution, which can also be applied to bioessential materials, is the cumulative availability curve method (Tilton et al. 2018; Tilton & Lagos 2007; Yaksic and Tilton 2009). This refers to the concept of the opportunity cost paradigm. A cumulative availability curve shows the total quantities of a commodity that can be produced economically at various prices with today’s technology and other conditions, e.g., environmental or social constraints and when including environmental costs. As discussed above, as price rises, reserves grow, usually nonlinearly higher with decreasing cut-off points (i.e., minimum economic concentration); the same applies to deposits.

**Fig. 3:**
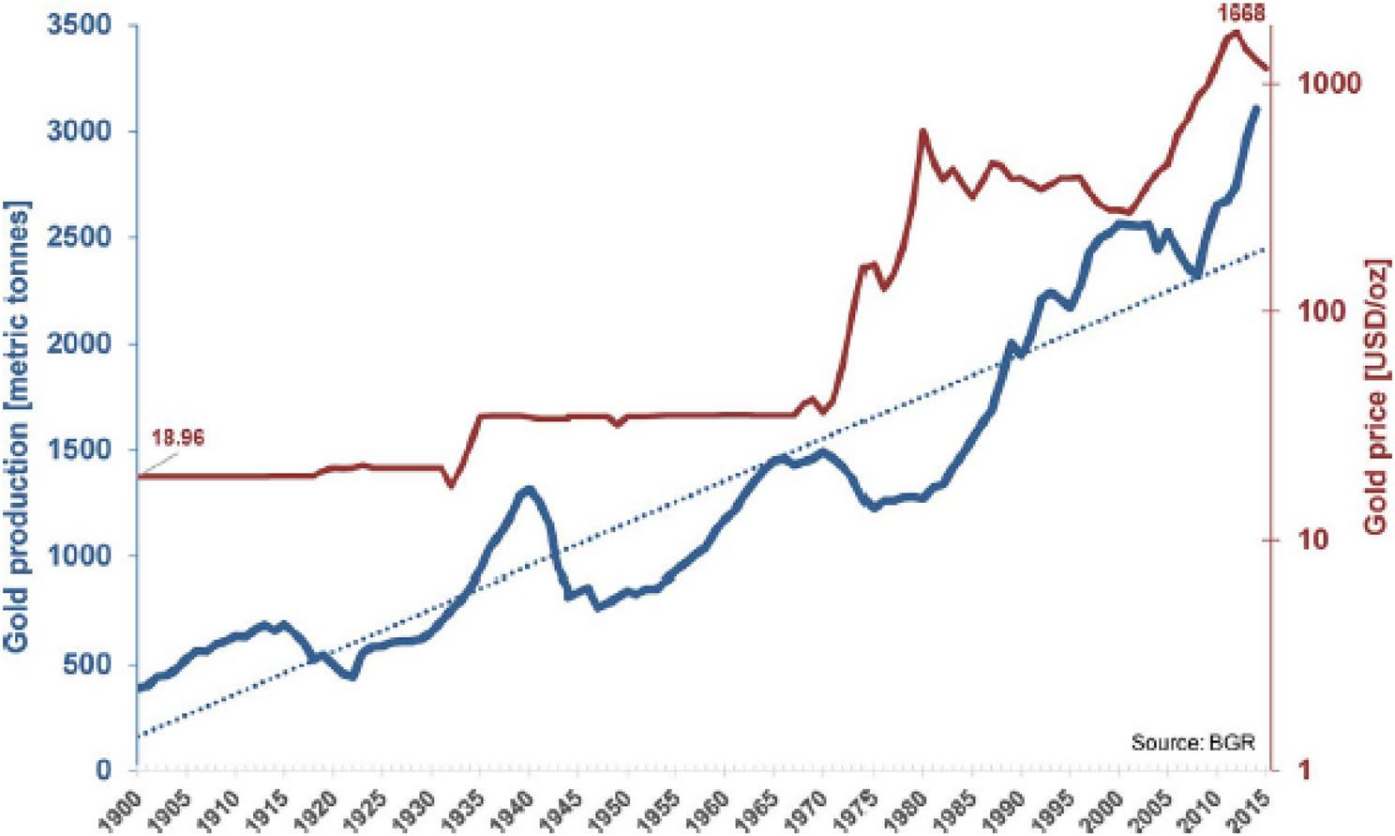
Gold production worldwide and gold price development since 1900 (nominal prices in logarithmic scale; source BGR Databank 2016) (Wellmer and Scholz 2018)

Let us consider how these functions. Deposits are enrichments in the Earth’s crust. Consequently, there are limits to the amounts that can be exploited in a defined cost and price frame. Because of these limits, whether they are geoeconomic (e.g., cut-off boundaries), geographic, or other limitations, curves of production histories (Wellmer and Scholz 2017) generally show a typical bell-shaped development over time, i.e., the Hubbert curve described above. If prices rise, another collective of deposits will become economically feasible, either discovered in the geopotential field or by the process by which, thus far, uneconomic resources become reserves. For this collective of deposits, over its lifetime another bell-shaped curve will develop. An example for such a development is the case history of gold since 1900 shown in Fig. 3 (Wellmer and Scholz 2018). Due to new price plateaus three production peaks developed (in the logarithmic presentation), i.e. peaks of overlapping Hubbert curves. Only the fourth peak in 1940 was caused by war measures.

We argue that there is no reason for pessimism about the availability of natural resources for a long time, even for bioessential ones, and that the fixed-stock concept is flawed. This may be considered a cornucopian view. Yet, we have to ask when this assumption may fail (falsifiability). We focus on phosphorus for which scarcity doubts are most frequently raised. One reason why the positive view may fail is the development of the reserves and resources; these are sociogeologic entities. Their magnitude depends on human ability to gain access to favourable areas and to have available the right technology for exploitation.

Technological development is difficult to forecast. Thus, conservative planning should rely on the technologies already available. Naturally, technology innovation for deep mining (below 1500 m) or environmentally friendly deep-sea mining would open huge phosphorus resources. The only possibility is the close monitoring of the longer time series of the reserve data and of the reserve/consumption (R/C) ratio, which may serve as an early-warning indicator (Scholz and Wellmer 2013).

Availability of phosphorus assessments have to acknowledge changes on the demand. One factor are the current total and agricultural use efficiencies. These are critically low (i.e., below 5%; Scholz and Wellmer 2015b) and certainly must be improved.

Because of the bioessentiality of phosphorus in the international phosphorus community, the necessity for improving the resource data has been voiced (Van Vuuren et al. 2010). Establishing an international organization for this task has already been mentioned (Rosemarin and Jensen 2013). Wellmer and Scholz (2015) made concrete proposals for such an organization under the auspices of the International Union of Geological Sciences, or EuroGeoSurveys, the Association of European Geological Surveys. This proposal was considered and subsequently published by the German science academies in 2018 (Leopoldina 2018).

## Discussion

### Ways of endangering science integrity

There are many ways to damage the integrity of science, such as fraud (falsifying and surpressing data; see e.g., George and Buyse 2015; Pickett and Roche 2018); sloppy experimentation and replication (Ioannidis 2005; Koshland 1987); plagiarism; (internal or external) censorship; and greed related to funding or remuneration or to career promotion (Ioannidis 2005; Saltelli et al. 2016; Woolf 1986). This paper deals with endangering the reputation and integrity of science when applying scientific knowledge on mineral resources scarcity. This is an example for a complex, societally relevant real-world problem of sustainable development. We have presented and discussed three critical factors that have the potential to endanger the integrity of science in this context.

First, there is increasing societal and political pressure for science to become purposeful and to solve problems (solutionism). Such knowledge becomes capitalized to finance research. This third mission may open the door to advocacy. The presentation and even the generation of data and knowledge may become biased for non-scientific reasons, and scientists may become social activists and policy advocates. This implies science communication in a more or less unbalanced way.

Second, some scientists and science activists claim that scientific findings, models, and theories may be applied as proven and indisputable and, thereby, be granted the status of becoming unfalsifiable*.* This holds true even for applications to complex systems for which any model or theory is genuinely incomplete*.* In regard to climate research, this truth to power has also turned to consensus to power (Van der Sluijs 2012). Herein, we have called this Faktengewalt; scientific findings are becoming facts that may not be questioned.

Third*,* in some opposition to Faktengewalt, post-normal science doubts that science may provide validated results that may refer to complex real-world problems. Post-normal scientists also stress that misconduct and fraud induce a principally biased knowledge system (Saltelli et al. 2016). Naturally, this endangers the integrity of science too. A main function of science, i.e., that science may serve as a (widely independent) clearinghouse of knowledge*,* becomes questioned. As an impact, science becomes just one voice among other stakeholder voices.

### The promotion of fallacious scarcity claims

The claim of scarcity of global resources is of political and societal interest. It is a key assumption of strong sustainability and a key statement to the Club of Rome that non-renewable minerals such as zinc, gold, and phosphorus will become scarce in a short- or mid-term time frame. With respect to phosphorus, the most cited paper on phosphorus management stated that “global reserves may be depleted in 50–100 years” (Cordell et al. 2009). In Sect. The phosphorus scarcity case, we have shown why it is fundamentally incorrect both from a conceptual and a mathematical modeling perspective.

In the paper by Cordell et al. (2009), as in most scarcity papers, the main error is that reserves and resources are considered as static, fixed-stock entities. The reserves, i.e., companies’ data for planning their businesses (together with the historically mined phosphate), are taken as a proxy for URR. The incorrect statement has been published in many premium newspapers and, thus, misleads political actors and the public. The result is that this misinformation has the potential to cause severe negative impacts from a sustainable development perspective, as investments in sustainability are made in the wrong places.

Reserves and resources are finite, but they are finite due to price levels, degree of exploration and technological capabilities. Limits can always be shifted by price rises and creativity improving technology. The reservoir, the geopotential, from where reserves can be generated is huge. the geological reservoir from which we can draw is practically limitless. A rough estimate of the total volume of phosphorus in Earth’s continental crust with a volume of about 7591 * 10^6^ km^3^ (Schubert and Sandwell 1989) and an estimate of average phosphorus pentoxide concentration (P2O5) of 0.027% (Binder 1999; P2O5 is used to assess rock phosphate volumes) provides a volume that would include the 2019 annual consumption of 0.24 Gt marketable phosphate rock (with 30% P2O5) for 68 million years. Copper is an excellent example of how close to the geochemical average value of Earth’s crust using our present technology (not considering future advances and improvements) we can get in exploitation. The lowest copper concentration producing mine of the world is the Aitik Mine in Sweden, mining 0.22% Cu with a cut-off grade of 0.06% Cu (Karlsson 2018). The cut-off is just 12 times the geological background of 50 ppm (Rankin 2011). Why should phosphate miners be less creative than copper miners if need arises?

Yet many scientists in the field of environmental and sustainability science continue to promote the scarcity claim based on an erroneous model. Recent statements read: “Our prognostication, based on the most recent estimates of reserves and taking appropriate consideration of projected demand levels, is that the peak in phosphorus production may occur between 2025 and 2084” (White & Cordell 2017, p. 62). In this statement, the year 2084 was calculated based on the updated reserves after the revision of the Moroccan reserves in 2010 (Jasinski 2011). Thus, the authors acknowledge a dynamics of what humankind may alternatively retrieve based only on the short-term changes in reserves, but exclude it from their predictive modeling. This is somewhat perplexing as the rectification of the falsity of the reasoning by Scholz and Wellmer (Scholz and Wellmer 2013) has been published in the same journal that has published the paper with the reserve-based global resources depletion. Some of the authors stopped mentioning global physical explicitly but resist making clarifying statements that would end the misleading reception of their incorrect claim.

### Why is the promotion of and belief in an incorrect scarcity model continuing?

There is a large range of cognitive, epistemological, motivational, and sociopolitical reasons for scientists to promote the wrong scarcity model outside mineral sciences. We present a discussion along three questions.

Q1: Is there a basic cognitive fallacy in non-mineral scientists’ reasoning that non-renewable, non-substitutable raw materials must become scarce in the course of human development?

Our answer is yes. There are several cognitive mechanisms causing fallacious reasoning. The one is that humans want assurance about the future of something they urgently need in concrete entities. Published data about reserves and resources or deposits in geographically well-confined areas fulfills this need. Thus, mines – and consequently reserves and resources – are conceived as static geologic entities that do not change. This is one root for the fixed-pie assumption. The much-discussed case of the Nauru Island guano deposit may serve as a prototypical cognitive reference (Déry and Anderson 2007). Mining of the seabirds’ excrement phosphate deposit on the surface of the 21 km^2^ island came to a rapid end. A second is that “humans have severe difficulties reasoning about magnitudes outside human perception” (Resnick et al. 2017). People have no intuitive cognitive access that the geological reservoir from which we can draw is practically limitless as shown above. And third, the ideas of practically unlimited possibilities that geologic enrichment processes, human creativity (McKelvey 1972), technological developments, and the stimulus of raw material prices driven by demand and supply offer opportunities to find solutions to meet humans’ needs are not part of the daily mindset.

A second cognition-related question is that reserves and resources are a dynamic geoeconomic, sociotechnological entity, as shown above.

Q2: What are the reasons that the dynamic nature of resources and reserves is often ignored?

What parts of terrestrial Earth may become inaccessible due to urbanization or environmental regulation depends on social constraints. What ore grades may be economically mined depends on the price society is able and willing to pay for one ton of a mineral. Yet this should be consistent with the intuitive mindset of limited numbers of deposits, i.e., mineral accumulations. Even the masters of environmental system dynamics modeling, Dennis and Donella Meadows, failed to recognize the geoeconomic rule of prices in relation to the increase of reserves/resources. They modeled a collapsing world, due also to mineral scarcity, also due to underestimation of the reserves because of incomplete exploration. Their focus has been exclusively on the demand side. Reserves and resources may be viewed as prototypical examples that the interaction or coupling of human systems dynamics and environmental systems dynamics is not well understood at the current stage of human development. The feedback cycle of reserves and resources, including the fact that growing global consumption is increasing the reserves and resources, seems to continue to be counterintuitive.

Thus far, we have argued from a cognitive perspective. Yet the scarcity fallacy may also be viewed from a history of science and epistemological perspective. Relating knowledge, variables, and dynamics that form social and natural systems is not meeting the traditional formation of disciplines and scientific communities. Knowledge, also in the minds of scientists, is often applied in specific contexts. For instance, for geologists it is clear that – in general – there is a non-linear increase of total tonnage of phosphate or other minerals with the decrease in the concentration of phosphate rock. Thus, reserves and resources increase non-linearly. This again has a positive effect on the price increase resources rule from a potential (long-term) scarcity management view. A lack of geologic literacy (with respect to time and space) and an understanding that reserves and resources are fundamental dynamic geoeconomic or geosocial entities and not fixed materials may also promote the prevalence of the “fixed-pie model.”

For answering Q1 and Q2, empirical research may clarify whether a lack of geologic literacy (with respect to time and space) and an understanding that reserves and resources are fundamental dynamic geo-economic or geosocial entities and not fixed materials promote the prevalence of the “fixed-pie model.” Answering this question may well become a subject of cognitive fallacy research clarifying misconceptions and biases regarding mineral-resource systems.

Finally, a key question related to scientists' motivation asks:

Q3: Are (some) scientists promoting the short- and mid-term scarcity fallacy because of sociopolitical motivations?

As with the other questions, systematic research to find the answer is missing. Environmental concern or advocacy about overuse of phosphorus and its environmental impacts (see 2.3) and the science activist’s and solutionist’s advocacy habit (see 2.3) may be effectively used to promote the wrong scarcity assumption as a way of reinforcing the argument for reducing losses and increasing phosphorus recycling. Here, an incorrect scientific statement about future reserves and resources is used as Faktengewalt. The motivation or policy-based habit may be viewed as serving the public with good and sustainable action. Yet, this may also result in just the opposite, i.e., setting the wrong priority among a range of different environmental investments due to an unjustified upgrading of the benefits of phosphorus recycling to avoid scarcity by fallacious reasoning. Incorrect scientific statements should be not abused for political action. In the course of a transdisciplinary project on Global Transdisciplinary Sustainable Phosphorus Management (Global Traps; Scholz et al. 2014) that included many practitioners, the authors noticed that participants from the phosphorus industry, mining companies and geologic surveys lost significant trust in the integrity of science based on the scarcity claim. Thus, the phosphorus scarcity claim was endangering partnerships with and the respect of those actors who not only have profound knowledge about the phosphorus geopotential, but are also key agents of increasing resource efficiency. Mineral resources experts have unambiguous evidence that short- and mid-term scarcity will not take place. We may also argue that scarcity proponents suppress key data*.* Just consider the stance that the amount of geoecologic phosphorus resources – mostly not sufficiently explored so far – would provide 1200 times the consumption of the year 2019 (Jasinski 2020). We may assume that such an argument would alleviate any person’s scarcity fear of phosphorus running out in the next decades. Phosphorus may be scarce in some soils and phosphorus fertilizer too expensive and, thus, not sufficiently available to many farmers in developing countries. But the physical “Global Phosphorus Scarcity” (Cordell 2016), as it is still promoted in the titles of papers again and again (Alewell et al. 2020; Fillippelli 2021; Nanda et al. 2020), is a myth and misleading political argument. Given the essentiality of phosphorus and the genuine uncertainty around its geopotential, responsible sustainable action should be directed toward better understanding the constraints of long-term availability and mitigating environmental effects due to low efficiency, i.e., high losses, of phosphorus along the supply chain (Scholz and Wellmer 2015a, b).

## Conclusion

In the course of dealing with these complex demands, trust in scientists, confidence in scientific results, and the integrity of science have been and continue to be endangered. The present paper shows that this becomes critical if (a) science activism, solutionism, and normative transition advocacy are integrated and bias the scientific modeling. Abandoning disciplinary knowledge (e.g., the dynamics of entities such as reserves or resources) may be taken as example. This becomes critical when walking (b) the truth to power road and utilizing an unjustified Faktengewalt of science without reflecting on the incompleteness of knowledge. Perhaps as a consequence, (c) science’s societal role of serving as a clearinghouse of knowledge becomes doubted. The critical validation of scientific statements becomes doubted. Science becomes downgraded to just one voice among others.

We discussed the mineral scarcity fallacy for the case of phosphorus as a bio-essential mineral. We revealed fundamental errors in modeling related to a fixed-pie model of reserves and resources, utilized convergent validation by presenting different models and theories showing that there is no scarcity in physical phosphorus supply for a temporal magnitude of 1000 years or longer. We discussed reasons and motivations of (non-mineral) scientists for promoting the scarcity claim. The authors of this paper are aware that the incompleteness of knowledge also applies to the present authors’ reasoning. There may be new insights on the role of mineral fertilizers, patterns of igneous phosphorus deposits, new deep-mining technologies, etc. that may change the arguments. There are limits and constraints in our reasoning. The results are robust for a variation of the demand function of factor two and might be rethought for a fivefold demand. Yet utilizing a fixed-pie/stock assumption of entities such as reserves or resources does not align with the ideal of an honest knowledge broker who makes available the best and most accurate scientific knowledge for the best political decision.

Solutionism may lead to de-differentiation between the role and functions of scientists. Intrinsic solutionism, i.e., normative science activism, promotes biased or even erroneous scientific statements (Strohschneider 2014). The claim of short- or mid-term phosphorus scarcity has been presented as an example. Here, unambiguously wrong scientific (geoeconomic) modeling and statements on scarcity have been used to raise public concern about a collapsing food system. This is presumably done to promote more efficient phosphorus use and recycling, which is – from an environmental and sustainability perspective – a proper and reasonable consequence. Yet according to propositional logic, anything may be concluded from a wrong proposition. The potential costs of an incorrect phosphorus scarcity claim may lead to promoting overly expensive and (environmentally) inefficient phosphorus recycling technologies whereas ignoring investments with higher potential efficiencies at other stages of the supply chain or places that may achieve higher efficacies (Kraus et al. 2016; Morf et al. 2019). This seems obvious given the perceived impact of scarcity assertion on policy actors (see Table 2, No. 3). The wrong citations of the papers on scarcity are mostly made by researchers in papers on recycling or increasing use efficiency. Practitioners’ judgments, especially those from practitioners with a geological background, show a much higher reliability than those from phosphorus scientists from these domains.

Moreover, an erroneous scarcity statement may destroy trust in science. We have personally seen that many open-minded actors from the mineral industry community who turn away from “evidently unreliable science” with fierce comments (see Table 2, No. 4). This makes it difficult to address them about other sustainable innovations. Thus, not unsustainable action may be the major threat but the loss of integrity of science by using unvalidated or wrong scientific statements as Faktengewalt. Scientists as honest knowledge brokers should communicate what they know (and what they may not know), why, and how well they know it. This particularly requires that wrong claims become (authentically and uprightly) corrected.

## Supplementary Information

Below is the link to the electronic supplementary material.

Supplementary file1 (DOCX 233 KB)
