# Supplementary material for: Endangering the integrity of science by misusing unvalidated models and untested assumptions as facts: General considerations and the mineral and phosphorus scarcity fallacy
Source: Sustain Sci. 2021 Aug 26;16(6):2069–86. doi: 10.1007/s11625-021-01006-w (PMC8387211; doi:10.1007/s11625-021-01006-w)
Supplement: Supplementary file 1 — Supplementary file1 (DOCX 233 KB) [file 11625_2021_1006_MOESM1_ESM.docx]

Supplementary Information 1 to the Paper

**Endangering the integrity of science by misusing unvalidated models and untested assumptions as facts: General considerations and the mineral and phosphorus scarcity fallacy**

**Roland W. Scholz & Friedrich-W. Wellmer**

The below letter and questions were sent to 22 phosphorus practice experts which are listed at the below right side of the Global TraPs organizational chart from January 2013. The questionnaire was sent to 20 experts whose addresses could be identified. Two addresses of two members of geological surveys/mining companies could not be identified. We asked members of the US and the French geological survey to answer the questionnaire.

More details are described in the manuscript.

Dear colleague

We are writing to you because we need your answer to four brief questions. The background is a discussion among some colleagues from the domain of sustainability science of Fred and myself. The issue is **whether “a wrong phosphorus scarcity claim affect trust of practitioners in science in general and/or people working in sustainability science?”**

We would be very grateful if you could answer the below four questions

All answers will be dealt by absolutely confidential by. Nobody besides Fred Wellmer and Roland will see your answers. If you want to convey your personal statement (also to the public), please provide a comment at the end of this file.

Question 1:

Cordell et al. (2009) concluded in her well known paper:

 „… modern agriculture is dependent on phosphorus derived from phosphate rock which is a non-renewable resource and current global reserves may be depleted in 50–100 years. While phosphorus demand is projected to increase the expected global peak in phosphorus production is predicted to occur around 2030.“

Remark: This statement has never been corrected fundamentally (but just adjusted by updated reserves numbers) and is still widely cited.

Answer Q1 – Do you think that the statement is basically wrong?

(yes or no):

**Please only answer questions Q2-Q4 if you have answered this question with yes**

Question 2: Do you have the impression that in papers dealing with phosphate resources there is an increasing bias in the last 20 years to cite only papers which stress the limitations of phosphorous reserves/resources, neglecting papers which show the longevity and dynamic development of phosphorous reserves/resources?

Answer Q2

(yes or no):

Question 3: Do you have the impression that the “phosphorus scarcity soon” argument has influenced has influenced the political agenda of circular economy stressing recycling?

Answer Q2

(yes or no):

Question 4: Do you know people from your peer group, whose belief in the integrity of science got endangered by the wide spread of the wrong scarcity claim.

To all: Please provide a comment on what you think about the “we are running out of phosphorus soon” claim.

My comment:

You are allowed to cite this statement when using my name under the label “personal communication”.

(yes/no)

We thank you a lot and will you provide a feedback on the outcome of this short expert inquiry.

Roland Scholz and Fred Wellmer


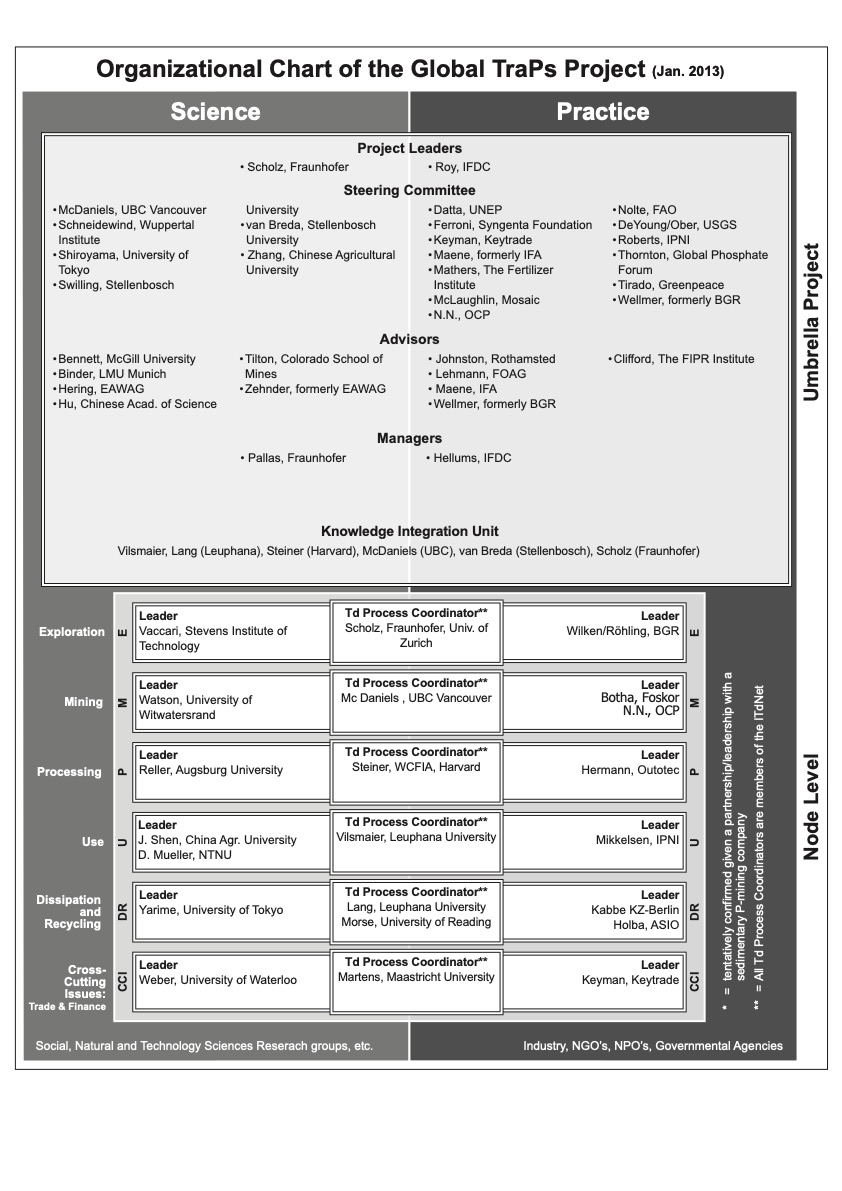


Cordell, D., Drangert, J. O., & White, S. (2009). The story of phosphorus: Global food security and food for thought. *Global Environmental Change-Human and Policy Dimensions, 19*(2), 292-305. doi:10.1016/j.gloenvcha.2008.10.009
